# Involvement of *aph(3′)-IIa* in the formation of mosaic aminoglycoside resistance genes in natural environments

**DOI:** 10.3389/fmicb.2015.00442

**Published:** 2015-05-19

**Authors:** Markus Woegerbauer, Melanie Kuffner, Sara Domingues, Kaare M. Nielsen

**Affiliations:** ^1^Integrative Risk Assessment – Data – Statistics, GMO Risk Assessment, Austrian Agency for Health and Food SafetyVienna, Austria; ^2^Faculty of Pharmacy and Center for Neuroscience and Cell Biology, University of CoimbraCoimbra, Portugal; ^3^Department of Pharmacy, University of TromsøTromsø, Norway; ^4^Genøk-Center for Biosafety TromsøTromsø, Norway

**Keywords:** antibiotic resistance, horizontal gene transfer, mosaic genes, homologous recombination, *npt*II

## Abstract

Intragenic recombination leading to mosaic gene formation is known to alter resistance profiles for particular genes and bacterial species. Few studies have examined to what extent aminoglycoside resistance genes undergo intragenic recombination. We screened the GenBank database for mosaic gene formation in homologs of the *aph(3′)-IIa* (*npt*II) gene. APH(3′)-IIa inactivates important aminoglycoside antibiotics. The gene is widely used as a selectable marker in biotechnology and enters the environment via laboratory discharges and the release of transgenic organisms. Such releases may provide opportunities for recombination in competent environmental bacteria. The retrieved GenBank sequences were grouped in three datasets comprising river water samples, duck pathogens and full-length variants from various bacterial genomes and plasmids. Analysis for recombination in these datasets was performed with the Recombination Detection Program (RDP4), and the Genetic Algorithm for Recombination Detection (GARD). From a total of 89 homologous sequences, 83% showed 99–100% sequence identity with *aph(3′)-IIa* originally described as part of transposon Tn*5*. Fifty one were unique sequence variants eligible for recombination analysis. Only a single recombination event was identified with high confidence and indicated the involvement of *aph(3′)-IIa* in the formation of a mosaic gene located on a plasmid of environmental origin in the multi-resistant isolate *Pseudomonas aeruginosa* PA96. The available data suggest that *aph(3′)-IIa* is not an archetypical mosaic gene as the divergence between the described sequence variants and the number of detectable recombination events is low. This is in contrast to the numerous mosaic alleles reported for certain penicillin or tetracycline resistance determinants.

## Introduction

Mosaic genes are genetic units consisting of DNA segments of different phylogenetic origin leading to sequence patterns which may confer novel phenotypic properties (Smith, 1992; Dowson et al., 1997; Boc and Makarenkov, 2011). The within gene (i.e., intragenic) recombination of DNA fragments increases the genetic plasticity of bacterial genomes and contributes to evolution and adaptability to new environmental conditions (Hanage et al., 2006). The process of mosaic gene formation primarily relies on the uptake of free DNA from the environment by competent bacteria via natural genetic transformation and subsequent integration of the incoming DNA fragment into the bacterial genome through homologous recombination (Smith et al., 1991). The efficiency of DNA segment integration is dependent on sequence similarity between the involved DNA strands. The frequency of homologous recombination decreases in a log-linear relationship with increasing sequence divergence between donor and recipient DNA to the point where it falls below the limit of detection—which is usually the case when pairwise sequence identity drops below 70% (Dowson et al., 1997; Fraser et al., 2007). This stringent similarity requirement may be circumvented by homology-directed illegitimate recombination, a mechanism where the integration of non-homologous DNA fragments is facilitated by the presence of a short homologous anchor sequence in the donor molecule and a region of microhomology on the opposite terminus of the incoming DNA with the target sequence (de Vries and Wackernagel, 2002; Prudhomme et al., 2002); or by double-illegitimate recombination, which is independent of any homology (Hulter and Wackernagel, 2008).

Genetic recombination inducing mosaic patterns in antibiotic resistance genes in bacterial pathogens results in therapy failure in clinical settings (Spratt, 1994; Heinemann and Traavik, 2004). Bacteria capable of lateral transfer of resistance gene fragments have the opportunity to evade selection pressure in response to alternating antibiotic therapy by acquiring new or modifying existing housekeeping genes and/or resistance determinants (Spratt, 1994). A prominent example is the mosaic pattern formation occurring in penicillin binding protein genes in *Streptococcus pneumoniae* (e.g., *pbp2b*) and *Neisseria* spp. (e.g., *penA*) and in tetracycline resistance determinants [e.g., *tet*(M), *tet*(O), *tet*(W)] in various animal and human pathogens (Spratt et al., 1989; Dowson et al., 1994; Patterson et al., 2007). These mosaic genes confer increased antibiotic resistance to the host bacterium and impact human health by increasing the morbidity and mortality rates of infectious diseases and by amplifying the financial burden of public health systems (Doern et al., 2001; Heinemann and Traavik, 2004; Bush et al., 2011).

An analysis of a potential contribution of the aminoglycoside resistance gene *aph(3′)-IIa* to the mosaic gene formation and the variability of *aph(3′)-II*-homologs is of relevance because this resistance gene is one of the most frequently applied selectable markers in genetic engineering and plant gene technology (Miki and McHugh, 2004; Shakya et al., 2011). Due to such technology applications this resistance gene is shed into the environment. Corresponding DNA fragments may additionally undergo chemical modifications when present as free extracellular DNA in the environment (Pontiroli et al., 2007; Chen et al., 2012). A recombination of anthropogenically released *aph(3′)-IIa* fragments with endogenous *aph(3′)-IIa* homologs present in competent environmental bacteria may lead to the formation of mosaic phosphotransferases with an altered antibiotic inactivation spectrum.

The enzyme APH(3′)-IIa inactivates the critically important aminoglycoside antibiotics neomycin and kanamycin as well as paromomycin, butirosin, gentamicin B, and ribostamycin (Shaw et al., 1993; WHO, 2012). Amikacin, a crucial second-line antibiotic used exclusively in humans, was shown to be phosphorylated to some extent only under *in vitro* conditions (Perlin and Lerner, 1986).

There is currently no experimental evidence available to support or disprove the hypothesis that antibiotic marker genes like *aph(3′)-IIa* may be involved in the formation of mosaic resistance genes. But powerful bioinformatic tools have now become available that allow *in silico* analysis of lateral intragenic gene transfer events (Boc et al., 2010; Martin et al., 2010; Boc and Makarenkov, 2011; Le et al., 2014).

To determine whether the genetic variability of *aph(3′)-IIa* like alleles available in GenBank has arisen from mosaic formation we performed a detailed *in silico* screening for intragenic recombination events in *aph(3′)-IIa* sequences utilizing phylogeny- and non-phylogeny-based algorithms of the Recombination Detection Program (RDP4) software package and the Genetic Algorithm for Recombination Detection (GARD) (Kosakovsky Pond et al., 2006a; Martin, 2010).

## Materials and methods

### Collection of sequence data

The *aph(3′)-IIa* gene from the *Escherichia coli* transposon Tn*5* (Accession number V00618, positions 151–945; 795 nts) was used as query sequence. This reference sequence termed for clarification “EcoAph3IIa” was searched against the bacterial non-redundant nucleotide collection (http://www.ncbi.nlm.nih.gov/nuccore/) and the database of reference genomic sequences (http://www.ncbi.nlm.nih.gov/refseq/). The discontiguous megablast algorithm was used with default settings except for 250 hits to be displayed (http://blast.ncbi.nlm.nih.gov/Blast.cgi?PROGRAM=blastn&PAGE_TYPE=BlastSearch&LINK_LOC=blasthome). Vectors, artificial sequences and models were excluded from the search. The search was carried out on September 22^nd^, 2014.

### Sequence alignments

Sequences producing BLAST matches were downloaded from GenBank, spanning the complete open reading frame when available. Multiple sequence alignments were prepared using the ClustalW algorithm implemented in Bioedit (http://www.mbio.ncsu.edu/bioedit/bioedit.html) (Hall, 2007). The sequence identity matrix option of Bioedit was used to determine the pairwise sequence identity between each sequence and the reference sequence *aph(3′)-IIa* (EcoAph3IIa). The sequence difference count matrix option of Bioedit was used to determine pairwise nucleotide differences among all aligned sequences.

### Selection of sequence sets for recombination analysis

All sequences sharing more than 60% sequence identity with the reference sequence EcoAph3IIa across their entire length were considered as “*aph(3′)-IIa* homologs.” Sequences with less than 60% sequence identity were considered as non-homologous. This distinction was based on the observation that *aph(3′)-IIb* (X90856) and *aph(3′)-IIc* (HQ424460), the closest described relatives of *aph(3′)-IIa* among aminoglycoside 3′-O-phosphotransferases (Ramirez and Tolmasky, 2010) share nearly 60% sequence identity with *aph(3′)-IIa*.

From the bulk of homologs collected from GenBank (Table 1), three sequence datasets were selected for recombination analysis:

**Table 1:** **The highest scoring BLAST hits for the** ***aph(3′)-IIa*** **gene from** ***E. coli*** **transposon Tn*****5***.

**Hit accession**	**Hit description (simplified)**	**Sequence length (nt)**	**Total identity (%)^a^**	**Short name (short name of representative)^b^**	**Data-set^c^**
**V00618**	***Escherichia coli* Tn*5* neomycin phosphotransferase (npt2)**	**795**	**100**	**EcoAph3IIa**	**3**
KC853434	*Escherichia coli* ACN001 plasmid pACN001-A	795	100	(EcoAph3IIa)	3
U32991	*Escherichia coli* mini-Tn*5* kanamycin transposon	795	100	(EcoAph3IIa)	3
X64335	*Escherichia coli* plasmid pMM234 DNA	795	100	(EcoAph3IIa)	3
AB255435	*Escherichia coli* plasmid pO86A1 DNA	795	100	(EcoAph3IIa)	3
L11017	*Escherichia coli* Tn*5* Tac1	795	100	(EcoAph3IIa)	3
U00004	*Escherichia coli* transposon Tn*5*	795	100	(EcoAph3IIa)	3
KJ747960	*Enterococcus faecalis* 3EH plasmid pCQ-3EH	795	100	(EcoAph3IIa)	3
CP000744	*Pseudomonas aeruginosa* PA7	795	100	(EcoAph3IIa)	3
AB366441	*Salmonella enterica* sv. Dublin pMAK2 DNA	795	100	(EcoAph3IIa)	3
JN983042	*Salmonella enterica* sv. Heidelberg pSH111_227	795	100	(EcoAph3IIa)	3
HF570109	*Shigella sonnei* plasmid pDPT3	795	100	(EcoAph3IIa)	3
JX469830	Uncultured bacterium plasmid pG527	795	100	(EcoAph3IIa)	3
NZ_JH724146	*Bacteroides dorei* CL02T12C06 supercont1.15	795	100	(EcoAph3IIa)	3
GQ463143	*Vibrio cholerae* Mex1 integrating conj.elem. ICEVchmex1	795	100	(EcoAph3IIa)	3
KF767856	*Salmonella enterica* sv. Typhimurium MRS_10/765 nptII	754	100		
**DQ449896**	**Uncultured bacterium clone K040 nptII–like gene**	**731**	**100**	**UncultK040**	**2**
**JQ664666**	***Riemerella anatipestifer* GN19 aph gene**	**717**	**100**	**RiemerGN19**	**1**
JQ664661	*Riemerella anatipestifer* GN12 aph gene	717	100	(RiemerGN19)	1
JQ664660	*Riemerella anatipestifer* GN10 aph gene	717	100	(RiemerGN19)	1
JQ664653	*Riemerella anatipestifer* FN3 aph gene	717	100	(RiemerGN19)	1
JQ664647	*Riemerella anatipestifer* 3 aph gene	717	100	(RiemerGN19)	1
JQ664646	*Riemerella anatipestifer* 1–5 aph gene	717	100	(RiemerGN19)	1
EF067857	*Escherichia coli* plasmid E99 aph	618	100		
NZ_GG698326	*Staphylococcus aureus aureus* TCH130 SCAFFOLD169	235	100		
NZ_GG698343	*Staphylococcus aureus* TCH130 SCAFFOLD186	66	100		
**AB702969**	***Escherichia coli* pCss165Kan: 4266 delta cssB::Km**	**795**	**99.8**	**Escheric03**	**3**
**DQ449901**	**Uncultured bacterium clone K047 nptII gene**	**728**	**99.8**	**UncultK047**	**2**
**DQ449899**	**Uncultured bacterium clone K048 nptII gene**	**728**	**99.8**	**UncultK048**	**2**
**DQ449898**	**Uncultured bacterium clone K001 nptII gene**	**728**	**99.8**	**UncultK001**	**2**
**JQ664680**	***Riemerella anatipestifer* X21-3N aph gene**	**717**	**99.8**	**RiemerX213**	**1**
**JQ664673**	***Riemerella anatipestifer* LQ30 aph gene**	**717**	**99.8**	**RiemerLQ30**	**1**
JQ664672	*Riemerella anatipestifer* LQ26 aph gene	717	99.8	(RiemerLQ30)	1
JQ664670	*Riemerella anatipestifer* GN52 aph gene	717	99.8	(RiemerLQ30)	1
JQ664668	*Riemerella anatipestifer* GN26 aph gene	717	99.8	(RiemerLQ30)	1
JQ664665	*Riemerella anatipestifer* GN18 aph gene	717	99.8	(RiemerLQ30)	1
JQ664664	*Riemerella anatipestifer* GN16 aph gene	717	99.8	(RiemerLQ30)	1
JQ664662	*Riemerella anatipestifer* GN13 aph gene	717	99.8	RiemerGN13	1
JQ664658	*Riemerella anatipestifer* GN5 aph gene	717	99.8	(RiemerLQ30)	1
**DQ449895**	**Uncultured bacterium clone K049 nptII gene**	**729**	**99.8**	**UncultK049**	**2**
**AF244993**	***Vibrio cholerae* aph3' gene**	**795**	**99.7**	**Vibrioch01**	**3**
**X57709**	***Escherichia coli* Transposon Tn*5* DNA for aphA-2 gene**	**795**	**99.7**	**Escheric02**	**3**
**NZ_DS995603**	***Clostridium nexile* DSM 1787 Scfld7**	**795**	**99.7**	**Clostrid01**	**3**
**DQ449903**	**Uncultured bacterium clone K002 nptII gene**	**728**	**99.7**	**UncultK002**	**2**
**DQ449900**	**Uncultured bacterium clone K003 nptII gene**	**728**	**99.7**	**UncultK003**	**2**
**JQ664676**	***Riemerella anatipestifer* LY37 aph gene**	**717**	**99.7**	**RiemerLY37**	**1**
**JQ664671**	***Riemerella anatipestifer* JN2N aph gene**	**717**	**99.7**	**RiemerJN2N**	**1**
**JQ664663**	***Riemerella anatipestifer* GN15 aph gene**	**717**	**99.7**	**RiemerGN15**	**1**
**JQ664659**	***Riemerella anatipestifer* GN9 aph gene**	**717**	**99.7**	**RiemerGN09**	**1**
**JQ664657**	***Riemerella anatipestifer* GN3 aph gene**	**717**	**99.7**	**RiemerGN03**	**1**
**JQ664655**	***Riemerella anatipestifer* GN1 aph gene**	**717**	**99.7**	**RiemerGN01**	**1**
**JQ664649**	***Riemerella anatipestifer* 8 aph gene**	**717**	**99.7**	**Riemer0008**	**1**
FN826652	Uncultured bacterium partial 16S rRNA gene US18.18	379	99.7		
**NZ_KB849231**	***Acinetobacter johnsonii* CIP 64.6 acLZl-supercont1.2**	**795**	**99.6**	**Acinetob01**	**3**
**DQ449897**	**Uncultured bacterium clone K036 nptII-like gene**	**729**	**99.5**	**UncultK036**	**2**
**JQ664678**	***Riemerella anatipestifer* W9 aph gene**	**717**	**99.5**	**RiemerW009**	**1**
**JQ664677**	***Riemerella anatipestifer* T2 aph gene**	**717**	**99.5**	**RiemerT002**	**1**
**JQ664675**	***Riemerella anatipestifer* LY18 aph gene**	**717**	**99.5**	**RiemerLY18**	**1**
**JQ664674**	***Riemerella anatipestifer* LY6 aph gene**	**717**	**99.5**	**RiemerLY06**	**1**
**JQ664667**	***Riemerella anatipestifer* GN22 aph gene**	**717**	**99.5**	**RiemerGN22**	**1**
**JQ664651**	***Riemerella anatipestifer* 256 aph gene**	**717**	**99.5**	**Riemer0256**	**1**
**JQ664650**	***Riemerella anatipestifer* 9 aph gene**	**717**	**99.5**	**Riemer0009**	**1**
**DQ449902**	**Uncultured bacterium clone K056 nptII gene**	**728**	**99.4**	**UncultK056**	**2**
**DQ449894**	**Uncultured bacterium clone K009 nptII-like gene**	**728**	**99.4**	**UncultK009**	**2**
**JQ664681**	***Riemerella anatipestifer* X23-4N aph gene**	**717**	**99.4**	**RiemerX234**	**1**
**JQ664679**	***Riemerella anatipestifer* X21-1N aph gene**	**717**	**99.4**	**RiemerX211**	**1**
**JQ664656**	***Riemerella anatipestifer* GN2 aph gene**	**717**	**99.4**	**RiemerGN02**	**1**
**JQ664654**	***Riemerella anatipestifer* FX2 aph gene**	**717**	**99.4**	**RiemerFX02**	**1**
**JQ664652**	***Riemerella anatipestifer* C6 aph gene**	**717**	**99.4**	**RiemerC006**	**1**
**JQ664648**	***Riemerella anatipestifer* 5 aph gene**	**717**	**99.4**	**Riemer0005**	**1**
**DQ449904**	**Uncultured bacterium clone K025 nptII-like gene**	**730**	**99.4**	**UncultK025**	**2**
V00615	Transposon Tn*5* left end	151	99.3		
**JQ664669**	***Riemerella anatipestifer* GN27 aph gene**	**717**	**99.2**	**RiemerGN27**	**1**
**CP001096**	***Rhodopseudomonas palustris* TIE-1**	**795**	**98.9**	**Rhodopse01**	**3**
GU721005	Uncult. Bact. plasmid clone mllc.F06 aph-like gene	197	98		
JQ937279	Uncultured bacterium aphA2 gene	347	97		
**KC543497**	***Pseudomonas aeruginosa* plasmid pOZ176**	**795**	**95.2**	**Pseudomo02**	**2**
NZ_KI519248	*Pseudomonas aeruginosa* U2504 adgfx-supercont1.7	795	91.9	(Pseudomo14)	3
NZ_KI519246	*Pseudomonas aeruginosa* U2504 adgfx-supercont1.5	795	91.9	(Pseudomo14)	3
**NZ_KI519240**	***Pseudomonas aeruginosa* U2504 adgfx-supercont1.1**	**795**	**91.9**	**Pseudomo14**	**3**
CP008824	*Enterobacter cloacae* ECNIH2 plasmid pKEC-39c	795	72.3	(Citrobac01)	3
CP008790	*Klebsiella oxytoca* KONIH1 plasmid pKOX-86d	795	72.3	(Citrobac01)	3
CP007732	*Klebsiella pneumoniae* KPNIH27 pKEC-dc3	795	72.3	(Citrobac01)	3
**CP007558**	***Citrobacter freundii* CFNIH1 plasmid pKEC-a3c**	**795**	**72.3**	**Citrobac01**	**3**
**HG938371**	***Burkholderia cenocepacia* H111 chromosome 2**	**795**	**65.9**	**Burkhold03**	**3**
**AM747721**	***Burkholderia cenocepacia* J2315 chromosome 2**	**795**	**65.5**	**Burkhold01**	**3**
**NZ_JH636049**	***Saccharomonospora xinjiangensis* XJ-54 Sacxiscaffold_2**	**792**	**64.5**	**Saccharo01**	**3**
**CP007509**	***Pseudomonas stutzeri* 19SMN4**	**795**	**63.8**	**Pseudomo13**	**3**
**CP000152**	***Burkholderia* sp. 383**	**795**	**63.6**	**Burkhold02**	**3**
CP007236	*Ensifer adhaerens* OV14 chromosome 1 sequence	795	58.6		
AY882987	*Sinorhizobium fredii* HH303-like gene	795	58.1		
CP001111	*Stenotrophomonas maltophilia* R551-3	804	51		
CP002585	*Pseudomonas brassicacearum* NFM421	795	49.3		
NZ_CM001512	*Pseudomonas fluorescens* Q8r1-96 chromosome	795	49.1		

a Total identities with the aph(3′)-IIa reference gene (EcoAph3IIa)

b Short names were given to sequences selected for further analysis. From groups of 100% identical sequences one representative was chosen for further analysis. Short names of representatives are indicated between parentheses and are given for all group members.

c Number of the respective dataset used for recombination analysis.

Search was performed against the non-redundant nucleotide collection and the database of genomic reference sequences. Bold sequences are unique variants.

Dataset 1: 36 partial sequences from the *Riemerella anatipestifer* isolate collection, representing the intra-species variation of *aph(3′)-IIa* homologs in a pathogen species residing in ducks (yellow bars in Figure S1).

Dataset 2: 11 partial sequences from river water, representing the variation of *aph(3′)-IIa* homologs occurring in bacterial species recovered from a defined natural aquatic environment (green bars, Figure S1).

Dataset 3: 34 full length *aph(3′)-IIa* homologs comprising the reference gene EcoAph3IIa and 33 sequences from various bacterial genomes and plasmids. This dataset represented the entire variation of *aph(3′)-IIa* genes known to date (i.e., as officially deposited in GenBank per September 22nd, 2014 (red, dark blue and light blue bars, Figure S1).

Each dataset was separately aligned with ClustalW and de-replicated to retain one representative sequence per variant. Pairwise differences among all variants were determined to allow selection of sequence subsets for improved recombination detection according to the recommendations of the instruction manual of RDP4 (Martin, 2010). It is indicated that RDP4 is unlikely to detect recombination between extremely similar sequences. The presence of multiple nearly identical sequences in a dataset unnecessarily increases the number of pairwise comparisons and the severity of multiple comparison correction and, thus, reduces sensitivity. The following formula was used for calculating the ratio between the number of sequences (X), length (L) and the minimum required pairwise distances (Y) in the dataset for sequences still eligible for recombination analysis by RDP4: Y = (2 × ln 4X) / L (Martin, 2010). On the other hand, highly divergent sequences increase the risk of false positives as they may cause misalignments and introduce an excess of variable sites into the alignment. Therefore, sequences sharing less than 70% sequence identities have to be handled with caution (Martin, 2010).

### Detection of recombination events in aligned sequence datasets

Recombination events in multiple sequence alignments were determined using the Recombination Detection Program Beta 4.36 package (RDP4). Seven of the recombination signal detection algorithms available as modules in RDP4 were employed: RDP (Martin, 2010), BootScan (Martin et al., 2005), MaxChi (Smith, 1992), Chimera (Posada and Crandall, 2001), GeneConv (Padidam et al., 1999), SiScan (Gibbs et al., 2000), and 3Seq (Boni et al., 2007). In the general settings for the RDP4 recombination detection procedure, the highest acceptable *p*-value was set to 0.05, the Bonferroni method was selected to correct for multiple comparisons and the entire process was run in permutational mode with 100 permutations. For the remaining parameters in the general RDP4 options defaults were retained. These defaults involved running PhylPro (Weiller, 1998) and LARD (Holmes et al., 1999) as secondary detection methods. Default settings were also retained for the options in the individual detection modules, except for MaxChi, where the specific window size was set to “variable.” These settings and analysis modules were chosen in accordance with common practice in literature (Keymer and Boehm, 2011; Smith et al., 2012; Thomas et al., 2012; Alvarez-Perez et al., 2013; Freel et al., 2013; Hester et al., 2013; Altamia et al., 2014; Duron, 2014).

Recombination breakpoints in multiple sequence alignments were confirmed with GARD (Kosakovsky Pond et al., 2006b) available at the datamonkey server (http://www.datamonkey.org/) using default values.

## Results

### Genetic diversity of *aph(3′)-IIa* homolog sequences in GenBank

The GenBank database was BLAST-searched for sequences similar to the *aph(3′)-IIa* gene from the transposon Tn*5* of *E. coli* (EcoAph3IIa). In total 227 hits were obtained. Table 1 summarizes the 94 highest scoring hits, and Figure S1 shows the regions of *aph(3′)-IIa* matched by these hits.

**Figure 1:**
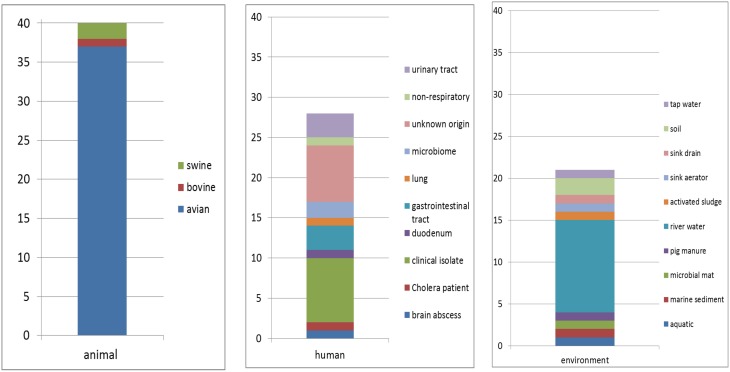
**Hosts and habitats of bacterial carriers of** ***aph(3′)-IIa*** **sequence variants**. Numbers of isolates are indicated.

**Figure 2:**
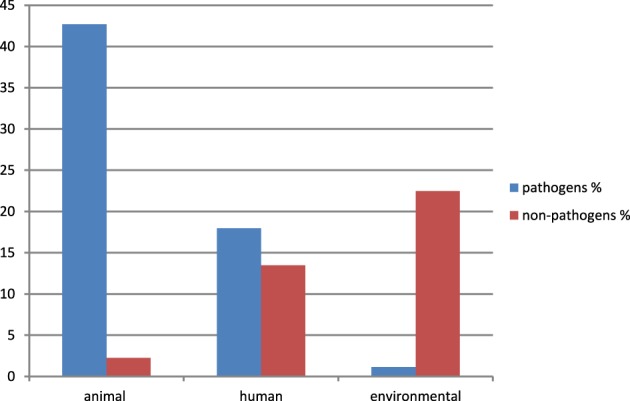
**Relative abundance and origin of bacterial isolates carrying** ***aph(3′)-IIa*** **variants**. Only isolates explicitly classified as “pathogen” in the GenBank entry or in one of its associated publications or showing a clear history as causative agents for disease as described in Murray et al. (1999), were considered as pathogens. All other isolates were identified as “non-pathogens” (including species characterized as opportunistic pathogens causing rare disease only in immunocompromised patients and “uncultured bacteria” without any additional information available). Data were calculated for a total of 89 isolates (=100%).

Eighty nine sequences showed sequence identities of 63–100% with EcoAph3IIa and were considered as *aph(3′)-IIa* homologs. Their bacterial carriers were of animal (40 isolates), human (28 isolates) and genuine environmental origin (21 isolates) (Figure 1). The large majority originated from avian hosts. Non-vertebrate samples were retrieved from such diverse environments as river water, soil, pig manure, activated sludge, marine sediments, and household installations (Figure 1). Most of the animal bacteria were pathogens (43%) but only a minimal fraction of the environmental isolates could be identified as causative agents for diseases (1%) (Figure 2). The *aph(3′)-IIa* gene sequence variant carriers comprised the following bacterial taxonomic classes: Actinobacteria, Alphaproteobacteria, Bacilli, Bacteroidia, Betaproteobacteria, Clostridia, Flavobacteria, and Gammaproteobacteria (Figure S2). Complete *aph(3′)-IIa* homologs had a length of 792–795 nts and discontiguous megablast produced alignment matches of 627–795 bp with the reference gene. Of these 89 homologs 26 were perfect 100% matches and 48 showed over 99% sequence identity with the reference sequence. The 99–100% BLAST matches included two sets of partial sequences originating from bacterial population surveys specifically targeting *aph(3′)-IIa* diversity: 36 sequences from isolates of the avian pathogen *R. anatipestifer* collected from diseased ducks (Yang et al., 2012), and 11 sequences from a cultivation independent monitoring of *aph(3′)-IIa* in Canadian river water samples (Zhu, 2007). As these sequences had been produced by PCR amplification with primers binding within the *aph(3′)-IIa* gene, sequence information was missing at their ends. Fifteen perfect (100%) and six nearly perfect (>99%) matches over the full gene length were detected in plasmid and genome sequences of bacteria phylogenetically as divergent as *E. coli*, *Bacteroides dorei*, and *Clostridium nexile*. The remaining six 99–100% matches represented gene fragments (66–754 nts; Table 1). Fifteen sequences were found to share 63–99% sequence identity with the reference sequence. These included two short sequence fragments from PCR-based studies on antibiotic resistance genes in water (JQ937279) and activated sludge (GU721005) and 13 complete genes from genomes and plasmids of *Pseudomonas aeruginosa*, *Enterobacter cloacae*, *Citrobacter freundii*, *Klebsiella pneumoniae*, *Klebsiella oxytoca*, *Saccharomonospora xinjiangensis, Pseudomonas stutzeri*, and *Burkholderia* spp. isolates (Table 1).

The remaining 136 hits shared only 44–59% sequence identity with EcoAph3IIa and, thus, were not considered as *aph(3′)-IIa* homologs. They included the *aph(3′)-IIc* gene of *Stenotrophomonas maltophilia* (HQ424460) and the *aph(3′)-IIb* (X90856) gene of *P. aeruginosa* (data not shown). The last sequence match presented in Table 1 and Figure S1 is an open reading frame of a *S. maltophilia* strain (CP001111) with 97% sequence identity to *aph(3′)-IIc*. These different aph genes varied in open reading frame length between 783 and 813 nts and produced discontiguous megablast matches spanning 50–370 bp between positions 360 and 720 of *aph(3′)-IIa*. The region between positions 360 and 720 of the *aph(3′)-IIa* gene contains two functional domains, known as motif1 and motif2, that are conserved across different clades of the aph gene family (Shaw et al., 1993).

### Sequence variation and recombination analysis in *aph(3′)-IIa* homologs from *Riemerella anatipestifer* isolates (dataset 1)

Of the 36 sequences from *R. anatipestifer* isolates, 25 were unique variants. One unique representative was selected from each group of identical sequences. The most frequent variant (RiemerGN19) was identical with the *aph(3′)-IIa* reference gene from the *E. coli* transposon Tn*5*. The sequences contained parts of the PCR primers used by the survey authors (Yang et al., 2012). After removal of the uninformative primer regions, a 686 nts gene segment, spanning *aph(3′)-IIa* between position 85 and 770 remained for recombination analysis. In total there were 45 polymorphic sites in the sequence alignment. Pairwise nucleotide differences ranged between 1 and 9 nucleotides. RDP4 analysis of the entire 25 sequence set did not reveal recombination signals. The analysis was repeated with a subset comprising the four most divergent sequences (RiemerX234, RiemerX211, RiemerFX02, RiemerC006). This subset corresponded to the recommendations of the RDP4 developers (Martin, 2010) regarding the relation between number, length and minimum divergence of the sequences. However, no recombination event was detected in this subset.

### Sequence variation and recombination analysis in *aph(3′)-IIa* homologs from river water (dataset 2)

All of the 11 *aph(3′)-IIa* sequences extracted from river water were unique variants. Sequence UncultK40 was identical with the *aph(3′)-IIa* reference gene (EcoAph3IIa). After removal of PCR primer binding sites, a 688 nts gene segment, spanning *aph(3′)-IIa* between position 27 and 714 remained for recombination analysis. Pairwise nucleotide differences ranged between 1 and 9 nucleotides. RDP4 did not detect recombination events neither in the complete set of 11 sequences, nor in the alignment of the three most divergent sequences (Uncultk56, UncultK009, UncultK025).

### Sequence variation and recombination analysis in full length *aph(3′)-IIa* homologs from various bacterial genomes and plasmids (dataset 3)

**Figure 3:**
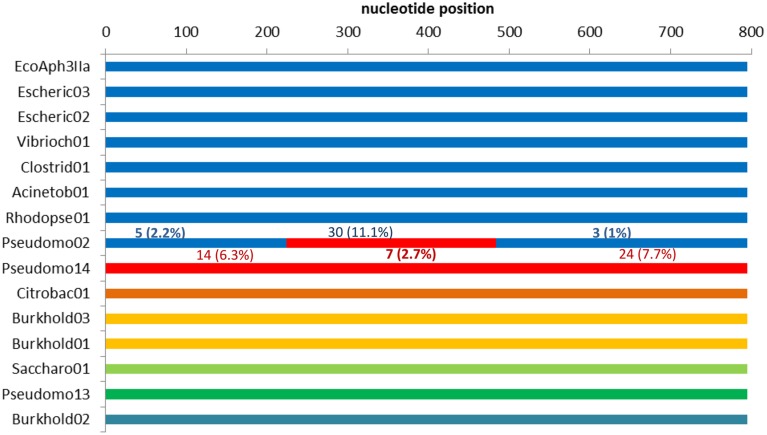
**Detection of a recombination event in dataset 3 (15 full length** ***aph(3′)-IIa*** **homologs) by RDP4**. The colors indicate sequence similarity and the likely origins of the segments in the recombinant sequence. The upper seven sequences (depicted in blue) are close relatives but not identical. For each segment of the recombinant (Pseudomo02), the number of sites different from those in the corresponding regions of the proposed parents is indicated: differences to EcoAph3IIa are depicted in blue above the segment, differences to Pseudomo14 are given in red below the segment.

Of the 34 available full length homologs originating from various bacterial chromosomes and plasmids 15 were unique variants. The original *aph(3′)-IIa* gene (EcoAph3IIa) was representative for 15 sequences producing perfect BLAST matches. The three sequences from isolate *P. aeruginosa* U2504 were identical, and one was retained as representative (Pseudomo14). The *aph(3′)-IIa* homologs detected in plasmids of *E. cloacae*, *K. oxytoca, K. pneumoniae* and *C. freundii* were identical, and the sequence from *Citrobacter* was retained as representative for further analysis (Citrobac01). The 15 unique sequences comprised 795 nts, except for Sacharo01, which was one nucleotide triplet shorter. Pairwise sequence differences varied between 1 and 324 nucleotides. Seven recombination detection methods in RDP4 detected a single recombination event in this dataset (Table 2). The results suggested that Pseudomo02 was a mosaic of Pseudomo14 and a sequence highly similar to the reference sequence EcoAph3IIa (Figure 3). The seven methods congruently identified the exchange of a fragment in the region between alignment positions 100 and 500. Figure 3 visualizes the recombination event and highlights the recombination breakpoints at positions 224 and 484, which were proposed congruently by three different methods (Table 2). Analysis of a 5 sequence subset including only sequences with the recommended level of pairwise nucleotide differences (8–239 nts, for explanations see Materials and Methods) confirmed the results obtained with the complete 15 sequence dataset (Table 2). For further confirmation the 15 sequence set was analyzed with GARD. GARD analysis detected a single significant recombination breakpoint signal at position 198 (Table 3). Upon analysis of the five sequence subset, GARD produced several statistically non-significant breakpoint signals, including one at position 482.

Table 2**Detection of a recombination event in dataset 3 (15 full length** ***aph(3′)-IIa*** **homologs from various bacterial species) and in a subset of 5 sequences with RDP4**.

**Table:** 

**15 Sequence dataset**		**5 Sequence subset**	
**Sequences**	**Role in the recombination event**	**Sequences**	**Role in the recombination event**
EcoAph3IIa	Major Parent	EcoAph3IIa	Major Parent
Escheric03	Major Parent	Rhodopse01	Major Parent
Escheric02	Major Parent	Pseudomo02	Recombinant
Vibrioch01	Major Parent	Pseudomo14	Minor Parent
Clostrid01	Major Parent	Citrobac01	–
Acinetob01	Major Parent		
Rhodopse01	Major Parent		
Pseudomo02	Recombinant		
Pseudomo14	Minor Parent		
Citrobac01	–		
Burkhold03	–		
Burkhold01	–		
Saccharo01	–		
Pseudomo13	–		
Burkhold02	–

**Table:** 

**Detection method**	**Breakpoint positions**		***p*-Value**	**Breakpoint positions**		***p*-Value**
	**Begin**	**End**		**Begin**	**End**	
RDP	224	456	1.34E-02	224	456	2.64E-04
GENECONV	245	434	1.94E-02	275	434	4.26E-04
Bootscan	224	484	6.69E-03	224	484	1.22E-04
Maxchi	96^*^	552^*^	7.92E-06	185^*^	552^*^	1.18E-09
Chimera	99^*^	434^*^	2.71E-03	114^*^	485^*^	8.92E-09
SiSscan	224	484	3.49E-07	224	484	1.84E-08
PhylPro			NS			NS
LARD			NS			NS
3Seq	98	484^*^	2.99E-08	214	484	2.46E-09

* The actual breakpoint position is undetermined (it was most likely overprinted by a subsequent recombination event).

Minor Parent, Parent contributing the smaller fraction of sequence.

Major Parent, Parent contributing the larger fraction of sequence.

NS, No significant p-value was recorded for this recombination event using this method.

**Table 3:** **Confirmation of the recombination event in dataset 3 (15 full length** ***aph(3′)-IIa*** **homologs from various bacterial species) and in a subset of 5 sequences with GARD**.

	**Breakpoints**	**LHS *p*-value^a^**	**RHS *p*-value^b^**	**Significance^c^**
15 sequence dataset	198	8.80E-03	2.58E-02	^**^
5 sequence subset	32	1.50E-01	1.00E+00	N.S.
	348	2.28E-02	7.74E-01	N.S.
	482	2.80E-01	2.10E-02	N.S.

a LHS p-value that the partition left of this breakpoint has a topology different from that inferred from the partition on the right.

b RHS p-value that the partition right of this breakpoint has a topology different from that inferred from the partition on the left.

c Only breakpoints with both p-values < 0.05 are considered significant

** Significant; N.S, not significant.

## Discussion

Sequence analysis of antibiotic resistance genes coding for penicillin binding proteins or for tetracycline resistance determinants has revealed horizontal gene transfer events leading to mosaic gene formation (Dowson et al., 1994; Patterson et al., 2007). The aim of this work was to elucidate whether intragenic recombination also occurs in natural homologs of *aph(3′)-IIa* aminoglycoside resistance genes. To determine the natural variability of *aph(3′)-IIa* the GenBank database was screened for *aph(3′)-IIa* variants. The hits were subsequently analyzed for intragenic recombination signals with the RDP4 software package and the web-based tool GARD.

The analysis of the recombination potential of *aph(3′)-IIa* is of biological relevance because this resistance determinant is inactivating important aminoglycoside antibiotics like kanamycin and neomycin which are vital antimicrobial agents for veterinary purposes and in special cases for human therapeutic applications (WHO, 2012). Additionally, Aph(3′)-IIa was shown *in vitro* to be capable of extending its antibiotic inactivation spectrum to amikacin—an essential agent for the treatment of severe systemic infections caused by Gram negative bacteria and a crucial second-line antibiotic for combatting multidrug-resistant tuberculosis (Durante-Mangoni et al., 2009; WHO, 2011)–due to an exchange of a single amino acid (Kocabiyik and Perlin, 1992). Although a high-level *aph(3′)-IIa*-induced amikacin resistant phenotype was only demonstrated so far for an *E. coli* mutant laboratory strain that showed a reduced aminoglycoside uptake combined with a resistance gene amplification (Perlin and Lerner, 1986) these observations are indicative for a significant effect of *aph(3′)-IIa* sequence variability on the antibiotic resistance profile of this aminoglycoside phosphotransferase. Nevertheless, we are only aware of two studies dealing explicitly with *aph(3′)-IIa* sequence variations, both failing to provide a connection between genotype and antibiotic resistance phenotype or induced minimum inhibitory concentrations (MIC) (Zhu, 2007; Yang et al., 2012).

There are only a few studies available on the prevalence of *aph(3′)-IIa*. Shaw et al. reported 2.5% of all isolates resistant to kanamycin as carriers of *aph(3′)-IIa* (Shaw et al., 1993). Most of the remaining papers suggested a low abundance of this resistance determinant in natural habitats: *aph(3′)-IIa* was only rarely detected in bacterial isolates of human (Peirano et al., 2006; Woegerbauer et al., 2014) or environmental origin or in total soil DNA preparations (Leff et al., 1993; Smalla et al., 1993; Ma et al., 2011) or there was evidence of large seasonal fluctuations especially in river waters (Zhu, 2007). These findings indicate that i) bacterial *aph(3′)-IIa* carrier strains are available providing recombination partners for this resistance determinant and that ii) an artificial exposure of bacterial populations with *aph(3′)-IIa* copies from anthropogenic sources like laboratory waste discharges or antibiotic resistance marker gene carrying transgenic organisms–eventually in combination with aminoglycoside containing effluents or manure - may increase the likelihood for genetic recombination (Chee-Sanford et al., 2009; Chen et al., 2012).

BLAST search of GenBank revealed only a limited number of *aph(3′)-IIa* variants with sequence identities between 60 and 99%. This is in contrast to the many mosaic genes coding for penicillin binding proteins or tetracycline resistance determinants for which homologs with a continuous spectrum of sequence identity between 80 and 99% have been identified (Spratt, 1994; Oggioni et al., 1996; Hakenbeck, 2000; Hollingshead et al., 2000; Johansen et al., 2001; Prudhomme et al., 2002; Nakamura et al., 2012).

The retrieved *aph(3′)-IIa* sequence homologs comprised a wide range of variant sequences originating from a broad variety of environmental sources including soil, water, marine sediments, manure, sewage sludge, and diverse human (gut, skin, urinary tract, lung, brain) and animal habitats (birds, pigs, cows).

For recombination analysis, the *aph(3′)-IIa* homologs were grouped into 3 datasets originating from duck pathogens (dataset 1) and river water (dataset 2) as representatives for sequences from bacteria living in a common habitat with the obvious physical property to exchange gene fragments. The remaining unique full length *aph(3′)-IIa* homologs were from bacteria of diverse animal, human or genuine environmental origins which could not be allocated to a common biotope (dataset 3). A combined analysis of sequences from dataset 3 comprising such different ecosystems is valid since lateral transfer of fragments in the evolution of a gene of interest can be assessed by sequence comparison without the prerequisite that the source organisms are of the same species or have been isolated from a common habitat. For example Oggioni et al. discovered mosaic patterns in tetracycline resistance genes by comparing previously published sequences of tetracycline resistant *Enterococcus faecalis*, *S. pneumoniae*, *Staphylococcus aureus*, *Ureaplasma urealyticum*, and *Neisseria* spp. isolates (Oggioni et al., 1996). Similarly Boc et al. detected numerous recombination events in the evolution of the rubisco gene *rbc*L by comparison of amino acid sequences from various photosynthetic bacteria and algae (Boc and Makarenkov, 2011).

The theoretical lower limit for most of the RDP4 algorithms applied for the identification of a mosaic gene (i.e., a gene affected by intragenic recombination) is three (Martin, 2010). Many publications refer to approx. 8–12 sequences to be sufficient for a reliable identification of mosaic genes (Oggioni et al., 1996; Dowson et al., 1997; Filipe et al., 2000; King et al., 2005): Oggioni et al. used a total of eight sequences to identify *tet*(M) as mosaic gene *in silico* with high significance (Oggioni et al., 1996). Filipe et al. tested 12 *mur*M alleles (Filipe et al., 2000), Dowson et al. 8 pbp2b alleles (Dowson et al., 1997), and King et al. used 12 novel 5′ and 10 novel 3′ *nan*A alleles to establish gene mosaicism (King et al., 2005). Our efforts are far exceeding any data collections used so far for the detection of mosaic genes in a single approach.

In the analysis of our third dataset, seven sequence comparison algorithms of the RDP4 suite provided evidence for a recombination event. The risk of identifying false positives, i.e., of mistaking mutation for recombination events, is inherent to any *in silico* recombination detection strategy (Martin, 2010; Boc and Makarenkov, 2011). Therefore, it is current practice to confirm calculated recombination events with several methods, including phylogeny-based and substitution distribution-based algorithms (Bay and Bielawski, 2011; Boc and Makarenkov, 2011). The described recombination event in the *aph(3′)-IIa* gene dataset is supported by three phylogeny-based methods (RDP, BootScan, SiScan), 4 substitution distribution-based methods (MaxChi, GeneConv, Chimera, 3Seq) and to some extent also by the phylogeny-based genetic algorithm (GARD). In bacterial multi-locus sequence typing (MLST), a major application area of the RDP4 software, many authors have convened to accept a software reported recombination event, if it is detected by at least three methods with a Bonferroni-corrected *p*-value < 0.05 (Keymer and Boehm, 2011; Smith et al., 2012; Alvarez-Perez et al., 2013). This criterion is met by the recombination event described here. The different methods agreed on the exchanged gene region and on the recombination partners involved in this event, but proposed different positions as recombination breakpoints. This reflects the different aspects of information each algorithm is targeting in a sequence alignment (Martin, 2010).

The mosaic *aph(3′)-IIa* gene identified in our third dataset is located in a Tn*5* similar cassette on pOZ176, an incP-2 plasmid from the multidrug-resistant isolate *P. aeruginosa* PA96 (Xiong et al., 2013). Plasmid pOZ176 is of environmental origin showing homologies with a vector from the plant pathogen *P. fluorescens* and to genomic islands present in the environmental bacteria *Ralstonia solanacearum* and *Azotobacter vinelandii*. Codon usage analysis indicated that most of the resistance genes of pOZ176 were not originally from *P. aeruginosa* but acquired by horizontal gene transfer from other species indicating a long history of DNA rearrangements most probably driven by antibiotic selection (Xiong et al., 2013).

PA96 is reported to be phenotypically resistant to at least 13 antibiotics (including amikacin and gentamicin) from three different substance classes [ß-lactams (pencillins, cephalosporins, carbapenems), fluoroquinolones, and aminoglycosides] (Xiong et al., 2013). Whole genome sequencing revealed that PA96 is carrier of *aph(3′)-IIb* (Deraspe et al., 2014), which mediates resistance to kanamycin, neomycin, and butirosin (Zeng and Jin, 2003) potentially masking an antibiotic activity of the newly discovered *aph(3′)-IIa* mosaic gene. At present there is no information available whether this novel mosaic gene on pOZ176 is functionally active and expressing any antibiotic resistance phenotype.

Antibiotic resistance marker genes used in transgenic plants are in several cases plant-codon optimized versions of their bacterial counterparts (Roa-Rodriguez and Nottenburg, 2003). The plant-derived *aph(3′)-IIa* variant of the transgenic potato line EH92-527-1 (Amflora) contains a characteristic mutation. Alignment of the recombinant *aph(3′)-IIa* gene of pOZ176 with the plant-derived transgenic variant of *aph(3′)-IIa* from EH92-527-1 revealed an absence of the plant allele-specific mutation in pOZ176 and vice versa an absence of the mutations distinctive for the recombinant *aph(3′)-IIa* allele in the transgenic counterpart (data not shown due to confidential business information restrictions). These observations indicate that an involvement of this transgenic allele in the evolution of *aph(3′)-IIa* of pOZ176 is unlikely.

Compared to the complex recombination history of known mosaic genes such as *pbp2b* (Dowson et al., 1997), *murM* (Filipe et al., 2000), or *tet*(M) (Oggioni et al., 1996), the observed recombination frequency among *aph(3′)-IIa* homologs was low. Although intragenic recombination is thought to be a frequent process during bacterial evolution (Didelot and Maiden, 2010) our report is presenting the first evidence for only a single mosaic formation event among *aph(3′)-IIa* homologs. To verify the sensitivity of our approach, we analyzed sequence collections of *pbp2b*, *murM*, and *tet*(M) with RDP4 using the same settings, and detected a multitude of recombination breakpoints and corresponding *p*-values several orders of magnitude lower than those obtained with the *aph(3′)-IIa* datasets (data not shown). According to the currently available sequence information in GenBank and compared to typical mosaic genes *aph(3′)-IIa* appears to be less prone for intragenic recombination. However, it is important to realize that novel *aph(3′)-IIa* sequence variants becoming prospectively available may change the outcome of the *in silico* recombination analysis.

We conclude that a recombination event has occurred during the evolution of an *aph(3′)-IIa* homolog present on a plasmid of environmental origin in a pathogenic multi-resistant strain of *P. aeruginosa*. The observed number of variant *aph(3′)-IIa* sequences is low and their diversity appears to be not primarily driven by intragenic recombinations.

### Conflict of interest statement

The authors declare that the research was conducted in the absence of any commercial or financial relationships that could be construed as a potential conflict of interest.

## References

[B1] Altamia M. A. Wood N. Fung J. M. Dedrick S. Linton E. W. Concepcion G. P.. (2014). Genetic differentiation among isolates of *Teredinibacter turnerae*, a widely occurring intracellular endosymbiont of shipworms. Mol. Ecol. 23, 1418–1432. 10.1111/mec.12667 24765662 PMC4621784

[B2] Alvarez-Perez S. de Vega C. Herrera C. M. (2013). Multilocus sequence analysis of nectar pseudomonads reveals high genetic diversity and contrasting recombination patterns. PLoS ONE 8:e75797 10.1371/journal.pone.0075797 24116076 PMC3792982

[B3] Bay R. A. Bielawski J. P. (2011). Recombination detection under evolutionary scenarios relevant to functional divergence. J. Mol. Evol. 73, 273–286. 10.1007/s00239-011-9473-0 22210457

[B4] Boc A. Makarenkov V. (2011). Towards an accurate identification of mosaic genes and partial horizontal gene transfers. Nucleic Acids Res. 39, e144. 10.1093/nar/gkr735 21917854 PMC3241670

[B5] Boc A. Philippe H. Makarenkov V. (2010). Inferring and validating horizontal gene transfer events using bipartition dissimilarity. Syst. Biol. 59, 195–211. 10.1093/sysbio/syp103 20525630

[B6] Boni M. F. Posada D. Feldman M. W. (2007). An exact nonparametric method for inferring mosaic structure in sequence triplets. Genetics 176, 1035–1047. 10.1534/genetics.106.068874 17409078 PMC1894573

[B7] Bush K. Courvalin P. Dantas G. Davies J. Eisenstein B. Huovinen P.. (2011). Tackling antibiotic resistance. Nat. Rev. Microbiol. 9, 894–896. 10.1038/nrmicro2693 22048738 PMC4206945

[B8] Chee-Sanford J. C. Mackie R. I. Koike S. Krapac I. G. Lin Y. F. Yannarell A. C.. (2009). Fate and transport of antibiotic residues and antibiotic resistance genes following land application of manure waste. J. Environ. Qual. 38, 1086–1108. 10.2134/jeq2008.0128 19398507

[B9] Chen J. Jin M. Qiu Z. G. Guo C. Chen Z. L. Shen Z. Q.. (2012). A survey of drug resistance bla genes originating from synthetic plasmid vectors in six chinese rivers. Environ. Sci. Technol. 46, 13448–13454. 10.1021/es302760s 23215020

[B10] Deraspe M. Alexander D. C. Xiong J. Ma J. H. Low D. E. Jamieson F. B.. (2014). Genomic analysis of *Pseudomonas aeruginosa* PA96, the host of carbapenem resistance plasmid pOZ176. FEMS Microbiol. Lett. 356, 212–216. 10.1111/1574-6968.12435 24673340

[B11] de Vries J. Wackernagel W. (2002). Integration of foreign DNA during natural transformation of *Acinetobacter* sp. by homology-facilitated illegitimate recombination. Proc. Natl. Acad. Sci. U.S.A. 99, 2094–2099. 10.1073/pnas.042263399 11854504 PMC122324

[B12] Didelot X. Maiden M. C. (2010). Impact of recombination on bacterial evolution. Trends Microbiol. 18, 315–322. 10.1016/j.tim.2010.04.002 20452218 PMC3985120

[B13] Doern G. V. Heilmann K. P. Huynh H. K. Rhomberg P. R. Coffman S. L. Brueggemann A. B. (2001). Antimicrobial resistance among clinical isolates of *Streptococcus pneumoniae* in the United States during 1999–2000, including a comparison of resistance rates since 1994–1995. Antimicrob. Agents Chemother. 45, 1721–1729. 10.1128/AAC.45.6.1721-1729.2001 11353617 PMC90537

[B14] Dowson C. G. Barcus V. King S. Pickerill P. Whatmore A. Yeo M. (1997). Horizontal gene transfer and the evolution of resistance and virulence determinants in Streptococcus. Soc. Appl. Bacteriol. Symp. Ser. 26, 42S–51S. 10.1046/j.1365-2672.83.s1.5.x 9436316

[B15] Dowson C. G. Coffey T. J. Spratt B. G. (1994). Origin and molecular epidemiology of penicillin-binding-protein-mediated resistance to beta-lactam antibiotics. Trends Microbiol. 2, 361–366. 10.1016/0966-842X(94)90612-2 7850202

[B16] Durante-Mangoni E. Grammatikos A. Utili R. Falagas M. E. (2009). Do we still need the aminoglycosides? Int. J. Antimicrob. Agents 33, 201–205. 10.1016/j.ijantimicag.2008.09.001 18976888

[B17] Duron O. (2014). Arsenophonus insect symbionts are commonly infected with APSE, a bacteriophage involved in protective symbiosis. FEMS Microbiol. Ecol. 90, 184–194. 10.1111/1574-6941.12381 25041857

[B18] Filipe S. R. Severina E. Tomasz A. (2000). Distribution of the mosaic structured *murM* genes among natural populations of *Streptococcus pneumoniae*. J. Bacteriol. 182, 6798–6805. 10.1128/JB.182.23.6798-6805.2000 11073926 PMC111424

[B19] Fraser C. Hanage W. P. Spratt B. G. (2007). Recombination and the nature of bacterial speciation. Science 315, 476–480. 10.1126/science.1127573 17255503 PMC2220085

[B20] Freel K. C. Millan-Aguinaga N. Jensen P. R. (2013). Multilocus sequence typing reveals evidence of homologous recombination linked to antibiotic resistance in the genus Salinispora. Appl. Environ. Microbiol. 79, 5997–6005. 10.1128/AEM.00880-13 23892741 PMC3811353

[B21] Gibbs M. J. Armstrong J. S. Gibbs A. J. (2000). Sister-scanning: a Monte Carlo procedure for assessing signals in recombinant sequences. Bioinformatics 16, 573–582. 10.1093/bioinformatics/16.7.573 11038328

[B22] Hakenbeck R. (2000). Transformation in *Streptococcus pneumoniae*: mosaic genes and the regulation of competence. Res. Microbiol. 151, 453–456. 10.1016/S0923-2508(00)00170-4 10961458

[B23] Hall T. (2007). BioEdit Version 7.0.0. Available online at: http://www.mbio.ncsu.edu/bioedit/biodoc.pdf (Accessed August 14, 2014).

[B24] Hanage W. P. Fraser C. Spratt B. G. (2006). The impact of homologous recombination on the generation of diversity in bacteria. J. Theor. Biol. 239, 210–219. 10.1016/j.jtbi.2005.08.035 16236325

[B25] Heinemann J. A. Traavik T. (2004). Problems in monitoring horizontal gene transfer in field trials of transgenic plants. Nat. Biotechnol. 22, 1105–1109. 10.1038/nbt1009 15340480

[B26] Hester S. E. Park J. Goodfield L. L. Feaga H. A. Preston A. Harvill E. T. (2013). Horizontally acquired divergent O-antigen contributes to escape from cross-immunity in the classical bordetellae. BMC Evol. Biol. 13:209 10.1186/1471-2148-13-209 24067113 PMC3849452

[B27] Hollingshead S. K. Becker R. Briles D. E. (2000). Diversity of PspA: Mosaic genes and evidence for past recombination in *Streptococcus pneumoniae*. Infect. Immun. 68, 5889–5900. 10.1128/IAI.68.10.5889-5900.2000 10992499 PMC101551

[B28] Holmes E. C. Worobey M. Rambaut A. (1999). Phylogenetic evidence for recombination in dengue virus. Mol. Biol. Evol. 16, 405–409. 10.1093/oxfordjournals.molbev.a026121 10331266

[B29] Hulter N. Wackernagel W. (2008). Double illegitimate recombination events integrate DNA segments through two different mechanisms during natural transformation of *Acinetobacter baylyi*. Mol. Microbiol. 67, 984–995. 10.1111/j.1365-2958.2007.06096.x 18194157

[B30] Johansen B. K. Wasteson Y. Granum P. E. Brynestad S. (2001). Mosaic structure of Shiga-toxin-2-encoding phages isolated from *Escherichia coli* O157: H7 indicates frequent gene exchange between lambdoid phage genomes. Microbiology 147, 1929–1936. Available online at: http://mic.sgmjournals.org/content/147/7/1929.full.pdf 11429469 10.1099/00221287-147-7-1929

[B31] Keymer D. P. Boehm A. B. (2011). Recombination shapes the structure of an environmental *Vibrio cholerae* population. Appl. Environ. Microbiol. 77, 537–544. 10.1128/AEM.02062-10 21075874 PMC3020551

[B32] King S. J. Whatmore A. M. Dowson C. G. (2005). NanA, a neuraminidase from *Streptococcus pneumoniae*, shows high levels of sequence diversity, at least in part through recombination with *Streptococcus oralis*. J. Bacteriol. 187, 5376–5386. 10.1128/JB.187.15.5376-5386.2005 16030232 PMC1196044

[B33] Kocabiyik S. Perlin M. (1992). Altered substrate specificity by substitutions at Tyr218 in bacterial aminoglycoside 3′-phosphotransferase-II. FEMS Microbiol. Lett. 72, 199–202. 10.1016/0378-1097(92)90529-W 1324201

[B34] Kosakovsky Pond S. L. Posada D. Gravenor M. B. Woelk C. H. Frost S. D. (2006a). GARD: a genetic algorithm for recombination detection. Bioinformatics 22, 3096–3098. 10.1093/bioinformatics/btl474 17110367

[B35] Kosakovsky Pond S. L. Posada D. Gravenor M. B. Woelk C. H. Frost S. D. W. (2006b). Automated phylogenetic detection of recombination using a genetic algorithm. Mol. Biol. Evol. 23, 1891–1901. 10.1093/molbev/msl051 16818476

[B36] Le P. T. Pontarotti P. Raoult D. (2014). Alphaproteobacteria species as a source and target of lateral sequence transfers. Trends Microbiol. 22, 147–156. 10.1016/j.tim.2013.12.006 24461455

[B37] Leff L. G. Dana J. R. McArthur J. V. Shimkets L. J. (1993). Detection of Tn*5*-like sequences in kanamycin-resistant stream bacteria and environmental DNA. Appl. Environ. Microbiol. 59, 417–421. 8382021 10.1128/aem.59.2.417-421.1993 PMC202121

[B38] Ma B. L. Blackshaw R. E. Roy J. He T. (2011). Investigation on gene transfer from genetically modified corn (*Zea mays* L.) plants to soil bacteria. J. Environ. Sci. Health B 46, 590–599. 10.1080/03601234.2011.586598 21722080

[B39] Martin D. P. (2010). RDP3 Instruction Manual. Available online at: http://web.cbio.uct.ac.Eza/~darren/RDPManual.pdf (Accessed September 22, 2014).

[B40] Martin D. P. Lemey P. Lott M. Moulton V. Posada D. Lefeuvre P. (2010). RDP3: a flexible and fast computer program for analyzing recombination. Bioinformatics 26, 2462–2463. 10.1093/bioinformatics/btq467 20798170 PMC2944210

[B41] Martin D. P. Posada D. Crandall K. A. Williamson C. (2005). A modified bootscan algorithm for automated identification of recombinant sequences and recombination breakpoints. AIDS Res. Hum. Retroviruses 21, 98–102. 10.1089/aid.2005.21.98 15665649

[B42] Miki B. McHugh S. (2004). Selectable marker genes in transgenic plants: applications, alternatives and biosafety. J. Biotechnol. 107, 193–232. 10.1016/j.jbiotec.2003.10.011 14736458

[B43] Murray P. R. Baron E. J. Pfaller M. A. Tenover F. C. Yolken R. H. (1999). Manual of Clinical Microbiology, 7th Edn Washington, DC: ASM Press.

[B44] Nakamura K. Kohda T. Shibata Y. Tsukamoto K. Arimitsu H. Hayashi M.. (2012). Unique biological activity of botulinum D/C mosaic neurotoxin in murine species. Infect. Immun. 80, 2886–2893. 10.1128/IAI.00302-12 22665374 PMC3434576

[B45] Oggioni M. R. Dowson C. G. Smith J. M. Provvedi R. Pozzi G. (1996). The tetracycline resistance gene *tet*(M) exhibits mosaic structure. Plasmid 35, 156–163. 10.1006/plas.1996.0018 8812782

[B46] Padidam M. Sawyer S. Fauquet C. M. (1999). Possible emergence of new geminiviruses by frequent recombination. Virology 265, 218–225. 10.1006/viro.1999.0056 10600594

[B47] Patterson A. J. Rincon M. T. Flint H. J. Scott K. P. (2007). Mosaic tetracycline resistance genes are widespread in human and animal fecal samples. Antimicrob. Agents Chemother. 51, 1115–1118. 10.1128/AAC.00725-06 17178791 PMC1803118

[B48] Peirano G. Agerso Y. Aarestrup F. M. Dos Reis E. M. Dos Prazeres Rodrigues D. (2006). Occurrence of integrons and antimicrobial resistance genes among *Salmonella enterica* from Brazil. J. Antimicrob. Chemother. 58, 305–309. 10.1093/jac/dkl248 16782743

[B49] Perlin M. H. Lerner S. A. (1986). High-level amikacin resistance in *Escherichia coli* due to phosphorylation and impaired aminoglycoside uptake. Antimicrob. Agents Chemother. 29, 216–224. 10.1128/AAC.29.2.216 2424366 PMC176380

[B50] Pontiroli A. Simonet P. Frostegard A. Vogel T. M. Monier J. M. (2007). Fate of transgenic plant DNA in the environment. Environ. Biosafety Res. 6, 15–35. 10.1051/ebr:2007037 17961478

[B51] Posada D. Crandall K. A. (2001). Evaluation of methods for detecting recombination from DNA sequences: computer simulations. Proc. Natl. Acad. Sci. U.S.A. 98, 13757–13762. 10.1073/pnas.241370698 11717435 PMC61114

[B52] Prudhomme M. Libante V. Claverys J. P. (2002). Homologous recombination at the border: insertion-deletions and the trapping of foreign DNA in *Streptococcus pneumoniae*. Proc. Natl. Acad. Sci. U.S.A. 99, 2100–2105. 10.1073/pnas.032262999 11854505 PMC122325

[B53] Ramirez M. S. Tolmasky M. E. (2010). Aminoglycoside modifying enzymes. Drug Resist. Updat. 13, 151–171. 10.1016/j.drup.2010.08.003 20833577 PMC2992599

[B54] Roa-Rodriguez C. Nottenburg C. (2003). Antibiotic Resistance Genes and their Uses in Genetic Transformation, Especially in Plants. CAMBIA. Available online at: http://www.bios.net/daisy/Antibiotic/752.html

[B55] Shakya T. Stogios P. J. Waglechner N. Evdokimova E. Ejim L. Blanchard J. E.. (2011). A small molecule discrimination map of the antibiotic resistance kinome. Chem. Biol. 18, 1591–1601. 10.1016/j.chembiol.2011.10.018 22195561

[B56] Shaw K. J. Rather P. N. Hare R. S. Miller G. H. (1993). Molecular genetics of aminoglycoside resistance genes and familial relationships of the aminoglycoside-modifying enzymes. Microbiol. Rev. 57, 138–163. 8385262 10.1128/mr.57.1.138-163.1993 PMC372903

[B57] Smalla K. van Overbeek L. S. Pukall R. van Elsas J. D. (1993). The prevalence of *npt*II and Tn*5* in kanamycin-resistant bacteria from different environments. FEMS Microbiol. Lett. 13, 47–58. 10.1111/j.1574-6941.1993.tb00050.x

[B58] Smith J. M. (1992). Analyzing the mosaic structure of genes. J. Mol. Evol. 34, 126–129. 10.1007/BF00182389 1556748

[B59] Smith J. M. Dowson C. G. Spratt B. G. (1991). Localized sex in bacteria. Nature 349, 29–31. 10.1038/349029a0 1985260

[B60] Smith S. E. Showers-Corneli P. Dardenne C. N. Harpending H. H. Martin D. P. Beiko R. G. (2012). Comparative genomic and phylogenetic approaches to characterize the role of genetic recombination in mycobacterial evolution. PLoS ONE 7:e50070 10.1371/journal.pone.0050070 23189179 PMC3506542

[B61] Spratt B. (1994). Resistance to antibiotics mediated by target alterations. Science 264, 388–393. 10.1126/science.8153626 8153626

[B62] Spratt B. G. Zhang Q. Y. Jones D. M. Hutchison A. Brannigan J. A. Dowson C. G. (1989). Recruitment of a penicillin-binding protein gene from *Neisseria flavescens* during the emergence of penicillin resistance in *Neisseria meningitidis*. Proc. Natl. Acad. Sci. U.S.A. 86, 8988–8992. 10.1073/pnas.86.22.8988 2510173 PMC298417

[B63] Thomas J. C. Godfrey P. A. Feldgarden M. Robinson D. A. (2012). Draft genome sequences of *Staphylococcus aureus* sequence type 34 (ST34) and ST42 hybrids. J. Bacteriol. 194, 2740–2741. 10.1128/JB.00248-12 22535928 PMC3347186

[B64] Weiller G. F. (1998). Phylogenetic profiles: a graphical method for detecting genetic recombinations in homologous sequences. Mol. Biol. Evol. 15, 326–335. 10.1093/oxfordjournals.molbev.a025929 9501499

[B65] WHO (2011). Guidelines for the Programmatic Management of Drug-Resistant Tuberculosis - 2011 Update. Available online at: http://whqlibdoc.who.int/publications/2011/9789241501583_eng.pdf (Accessed Mar 4, 2013).

[B66] WHO (2012). Critically Important Antimicrobials for Human Medicine. 3rd Revision 2011. Geneva: WHO Press; World Health Organization. Available online at: http://www.who.int/foodborne_disease/resistance/cia/en/ (Accessed Oct 7, 2013).

[B67] Woegerbauer M. Zeinzinger J. Springer B. Hufnagl P. Indra A. Korschineck I. (2014). Prevalence of the aminoglycoside phosphotransferase genes *aph(3′)-IIIa* and *aph(3′)-IIa* in *Escherichia coli*, *Enterococcus faecalis*, *Enterococcus faecium*, *Pseudomonas aeruginosa*, *Salmonella enterica* subsp. enterica and *Staphylococcus aureus* isolates in Austria. J. Med. Microbiol. 63, 210–217. 10.1099/jmm.0.065789-0 24194558

[B68] Xiong J. Alexander D. C. Ma J. H. Deraspe M. Low D. E. Jamieson F. B.. (2013). Complete sequence of pOZ176, a 500-kilobase IncP-2 plasmid encoding IMP-9-mediated carbapenem resistance, from outbreak isolate *Pseudomonas aeruginosa* 96. Antimicrob. Agents Chemother. 57, 3775–3782. 10.1128/AAC.00423-13 23716048 PMC3719692

[B69] Yang F. F. Sun Y. N. Li J. X. Wang H. Zhao M. J. Su J.. (2012). Detection of aminoglycoside resistance genes in *Riemerella anatipestifer* isolated from ducks. Vet. Microbiol. 158, 451–452. 10.1016/j.vetmic.2012.02.027 22445728

[B70] Zeng L. Jin S. (2003). *aph(3′)-IIb*, a gene encoding an aminoglycoside-modifying enzyme, is under the positive control of surrogate regulator HpaA. Antimicrob. Agents Chemother. 47, 3867–3876. 10.1128/AAC.47.12.3867-3876.2003 14638496 PMC296182

[B71] Zhu B. (2007). Abundance dynamics and sequence variation of neomycin phosphotransferase gene (*npt*II) homologs in river water. Aquat. Microb. Ecol. 48, 131–140. 10.3354/ame048131

