# Supplementary figures and images for: Involvement of aph(3′)-IIa in the formation of mosaic aminoglycoside resistance genes in natural environments
Source: Front Microbiol. 2015 May 19;6:442. doi: 10.3389/fmicb.2015.00442 (PMC4437187; doi:10.3389/fmicb.2015.00442)

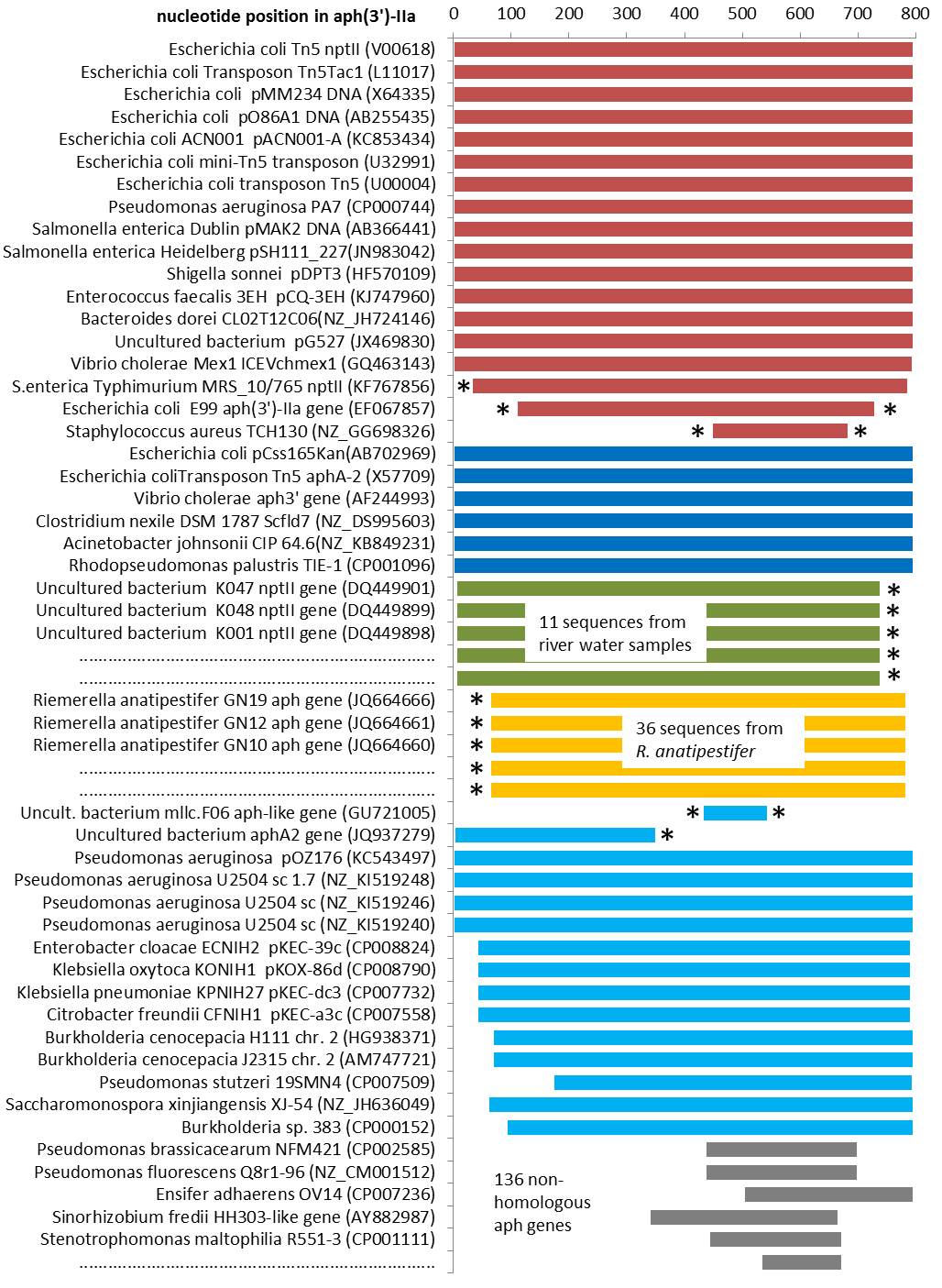

Supplement: Supplementary file 1 [file Image1.JPEG]

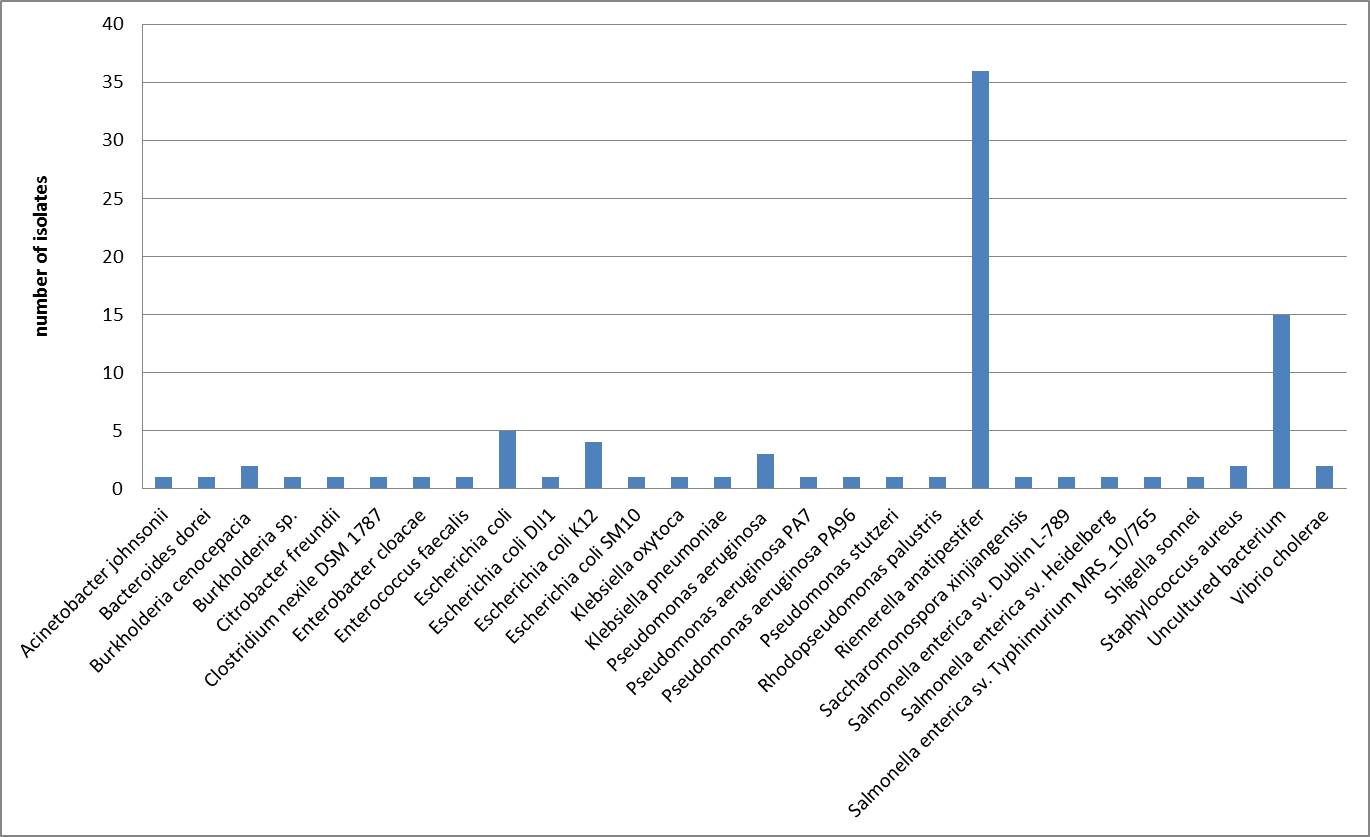

Supplement: Supplementary file 3 [file Image2.JPEG]
