# Supplementary material for: Involvement of aph(3′)-IIa in the formation of mosaic aminoglycoside resistance genes in natural environments
Source: Front Microbiol. 2015 May 19;6:442. doi: 10.3389/fmicb.2015.00442 (PMC4437187; doi:10.3389/fmicb.2015.00442)
Supplement: Supplementary file 2 [file Image1.PDF]

Supplementary Material

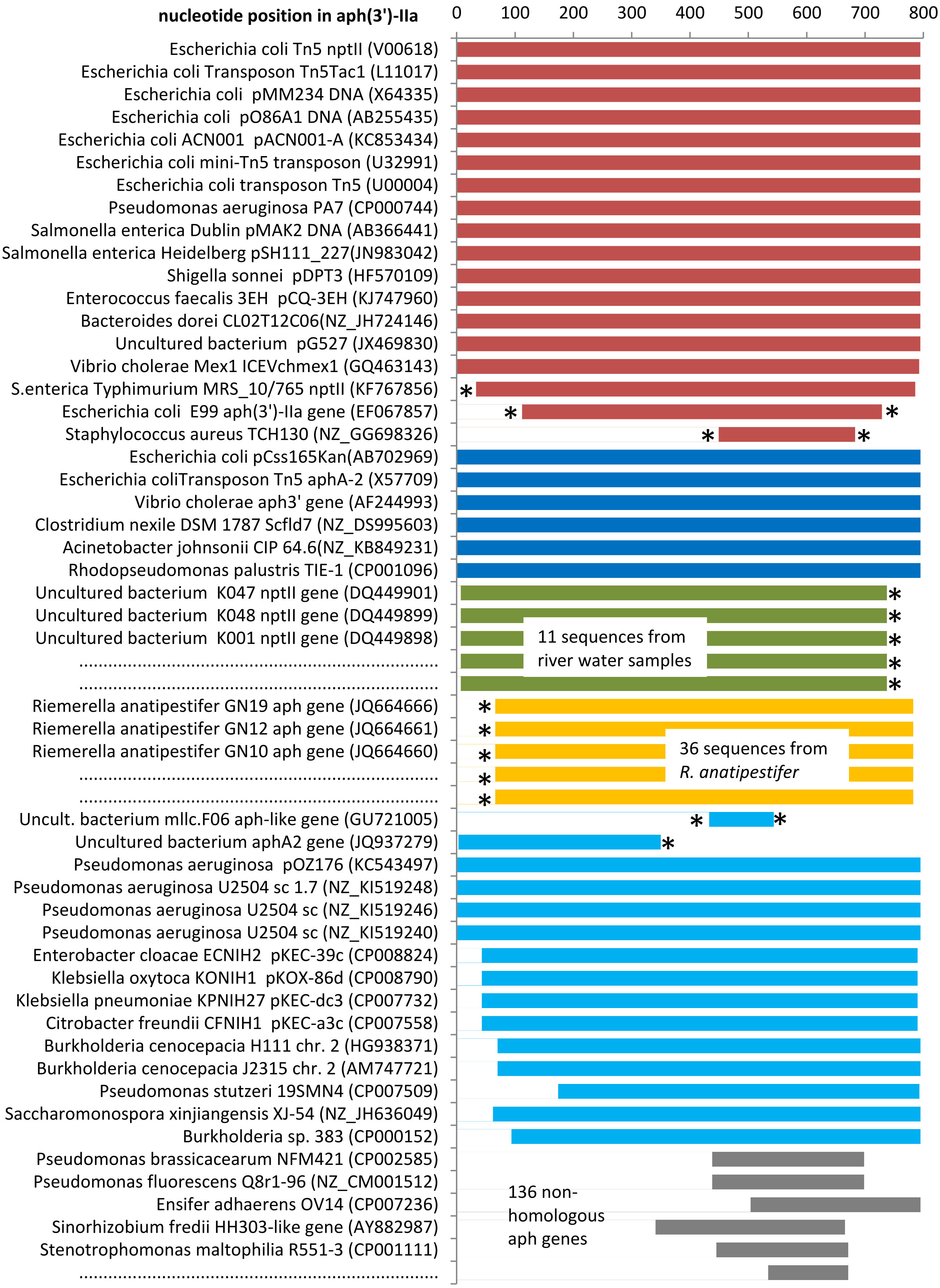

Figure S1. The highest scoring BLAST matches of the *aph(3')-IIa* gene with GenBank entries.

Color coding: red – sequences sharing 100% identity with the *aph(3')-IIa* reference sequence (dataset 3), dark blue – sequences sharing 99% to < 100% identity with *aph(3')-IIa* (dataset 3), light blue -sequences sharing 63% - 99% identity with *aph(3')-IIa* (dataset 3), yellow – partial sequences from a study on *Riemerella anatipestifer* isolates (Yang et al., 2012) sharing 99-100% sequence identity with aph (3')-IIa (dataset 1), green – partial sequences from a cultivation independent river water monitoring (Zhu, 2007), sharing 99% -100% identity with *aph(3')-IIa*, grey – non homologous aminoglycoside phosphotransferase genes sharing 44%-59% identity with *aph(3')-IIa*. *the asterisks mark partial sequences (fragments). Three fragments (NZ_GG698343, FN826652, V00615) are not shown.

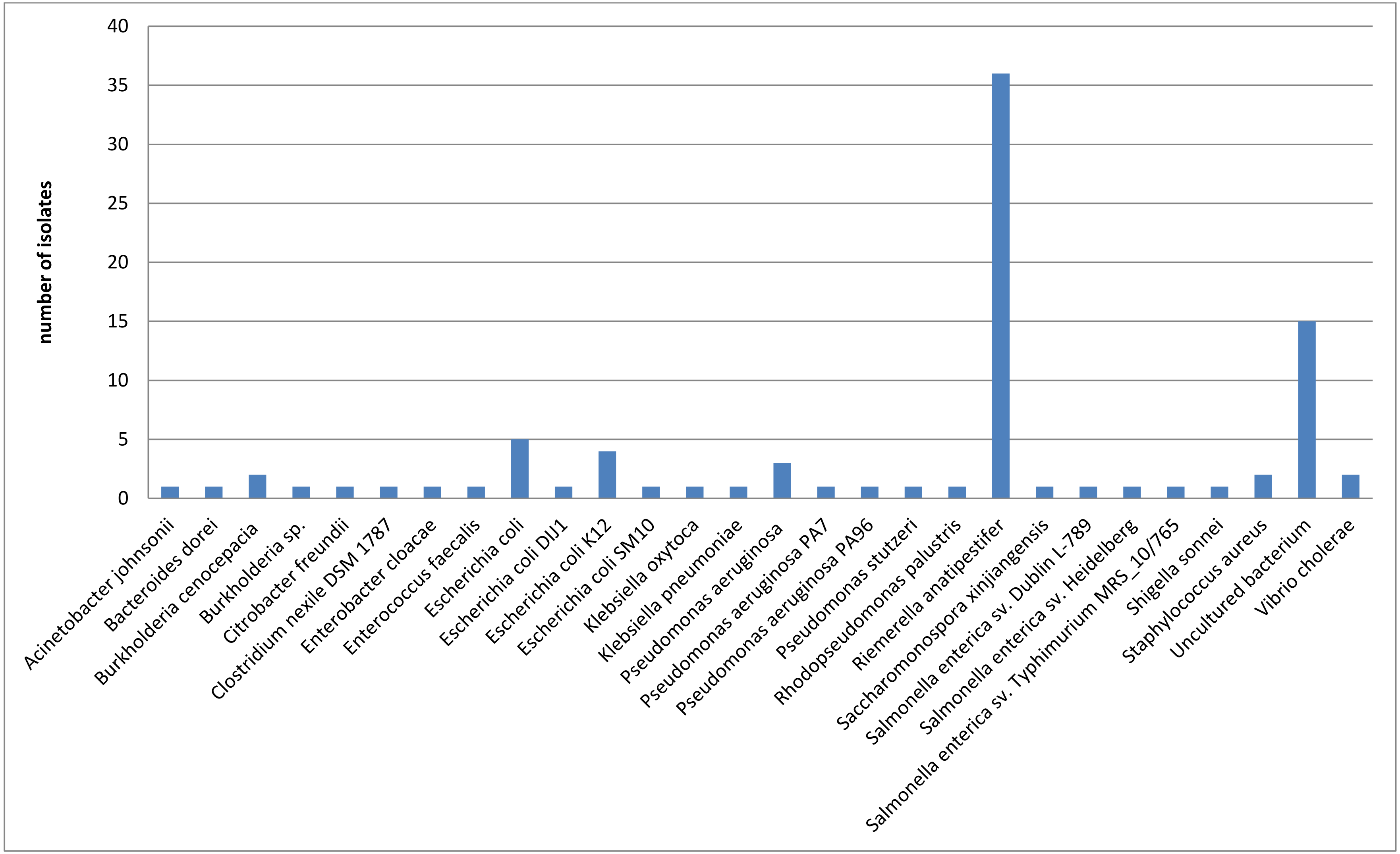

Figure S2. Number of isolates and bacterial species carrying *aph(3')-IIa* homologs considered in this study.
